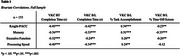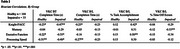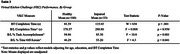# The Virtual Kitchen Challenge: Relations between everyday function and cognition in older adults with healthy or impaired cognition

**DOI:** 10.1002/alz70857_107704

**Published:** 2025-12-25

**Authors:** Kimberly Halberstadter, Marina Kaplan, Moira McKniff, Sophia L. Holmqvist, Molly B. Tassoni, Stephanie M. Simone, Riya Chaturvedi, Tania Giovanetti

**Affiliations:** ^1^ Temple University, Philadelphia, PA, USA

## Abstract

**Background:**

Subtle functional difficulties may reflect a breakdown in cognition and risk for future cognitive impairment. The Virtual Kitchen Challenge (VKC) is a non‐immersive virtual reality performance‐based measure of everyday function designed to efficiently measure subtle functional difficulties through tasks completed on a computer touchscreen. The present study examines relations between VKC performance and cognition among older adults with varying degrees of cognitive impairment.

**Method:**

133 community‐dwelling older adults classified with healthy or impaired cognition completed the VKC and cognitive assessments including the Knight Preclinical Alzheimer's Cognitive Composite (Knight‐PACC) and tests of memory, executive function, and processing speed. The VKC requires participants to make a breakfast and lunch (B/L) using objects on the touchscreen and includes a basic training (BT) trial to learn basic touchscreen movements. BT completion time and B/L completion time, percent task accomplishment, and percent time off‐screen were computed. Bivariate correlation, Mann‐Whitney U, and ANCOVA analyses were used to measure VKC‐cognition relations and differences in performance between healthy and impaired groups.

**Result:**

Better VKC performance was associated with higher cognitive function across all variables (*p*'s < .01). This pattern of associations was similar between healthy and impaired groups for VKC BT and B/L completion time variables; associations for B/L percent accomplishment and time off‐screen variables were stronger for the impaired group. In group analyses of VKC performance, BT completion time was higher for the impaired group (U=634, *p* < .01). After controlling for BT completion time, age, and education, the impaired group had lower task accomplishment (F=10.8, *p* < .01) and greater time off‐screen (F=4.3, *p* = .04), but there was no group difference for B/L completion time.

**Conclusion:**

VKC measures of task accomplishment and time off‐screen differed between healthy and impaired groups and may be sensitive to the cognitive aspects of everyday function at higher levels of impairment. Group differences in B/L completion time were eliminated after controlling for BT completion time, suggesting that VKC completion time may reflect motor or touchscreen proficiency. Future research should investigate VKC associations with computer proficiency and preclinical biomarkers, as well as VKC predictive validity for cognitive decline.